# Supplementary material for: Protein-protein interactions and metabolite channelling in the plant tricarboxylic acid cycle
Source: Nat Commun. 2017 May 16;8:15212. doi: 10.1038/ncomms15212 (PMC5440813; doi:10.1038/ncomms15212)
Supplement: Supplementary Information — Supplementary Figures and Supplementary Tables [file ncomms15212-s1.pdf]

Supplementary information

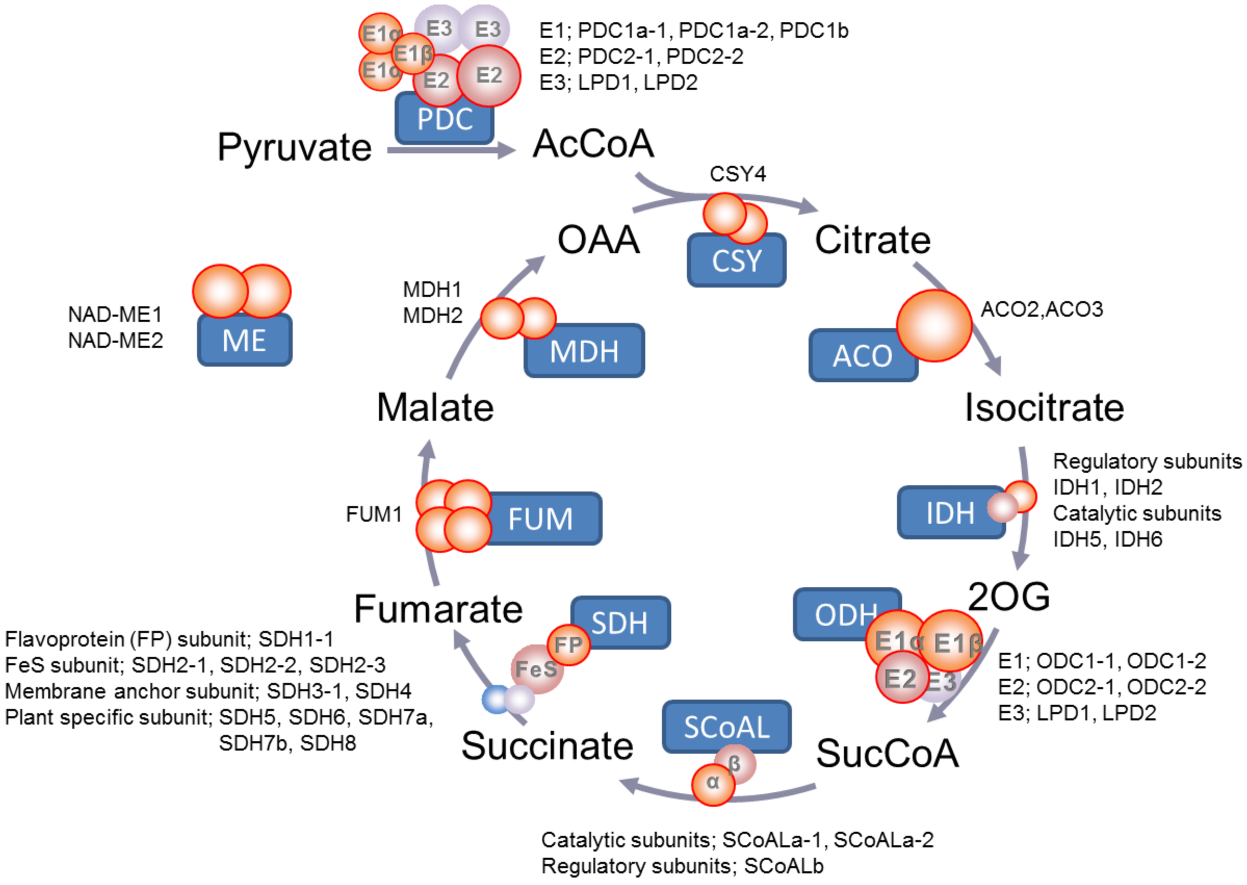

Supplementary Figure 1: Components of *Arabidopsis* tricarboxylic acid (TCA) cycle. Schematic summary of the TCA cycle and the enzymes related to the reactions. The large text and the arrows represent the intermediates and reactions, respectively. The enzyme names are shown in the blue rectangles. The subunits of the enzymes are represented by spheres with the size roughly proportional to the molecular mass of the protein. Differences in the shading colour represent the heteromeric nature of the enzymes. The names of subunit proteins are described as text beside the spheres. The spheres with red outlines are the subunits directly catalyse the conversion of TCA cycle intermediates. PDC, pyruvate dehydrogenase complex; ME, malic enzyme; CSY, citrate synthase; ACO, aconitase; IDH, isocitrate; ODC, oxoglutarate dehydrogenase complex; SCoAL, succinyl-CoA ligase; SDH, succinate dehydrogenase; FUM, fumarase; MDH, malate dehydrogense; AcCoA, acetyl-CoA; 2OG, 2-oxoglutarate; SucCoA, succinyl-CoA.

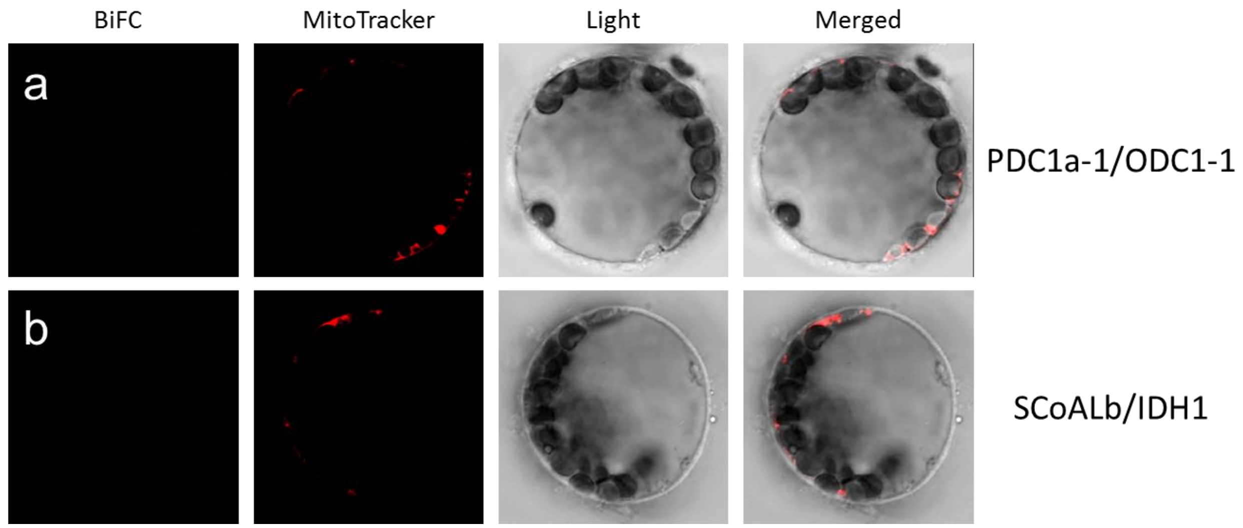

Supplementary Figure 2: Negative controls for bimolecular fluorescent complementation (BiFC) assay. Protein pairs which were not defined as interactors in any of three interaction assays were tested by BiFC with transient expression of tagged proteins in *Arabidopsis* mesophyll protoplasts. The panels from the left-hand show the BiFC fluorescence, fluorescence from MitoTracker orange staining, bright field image and the merged image of all of those, respectively. The scale bars represent 5 µm. The detail of the constructs can be found in Supplementary Table 6. a, PDC1a-1-SCYCE/ODC1-1-VYNE; b, SCoALb-SCYCE/IDH1-VYNE. Another negative control, FUM1-SCYCE/IDH1-VYNE, is found in Fig. 3.

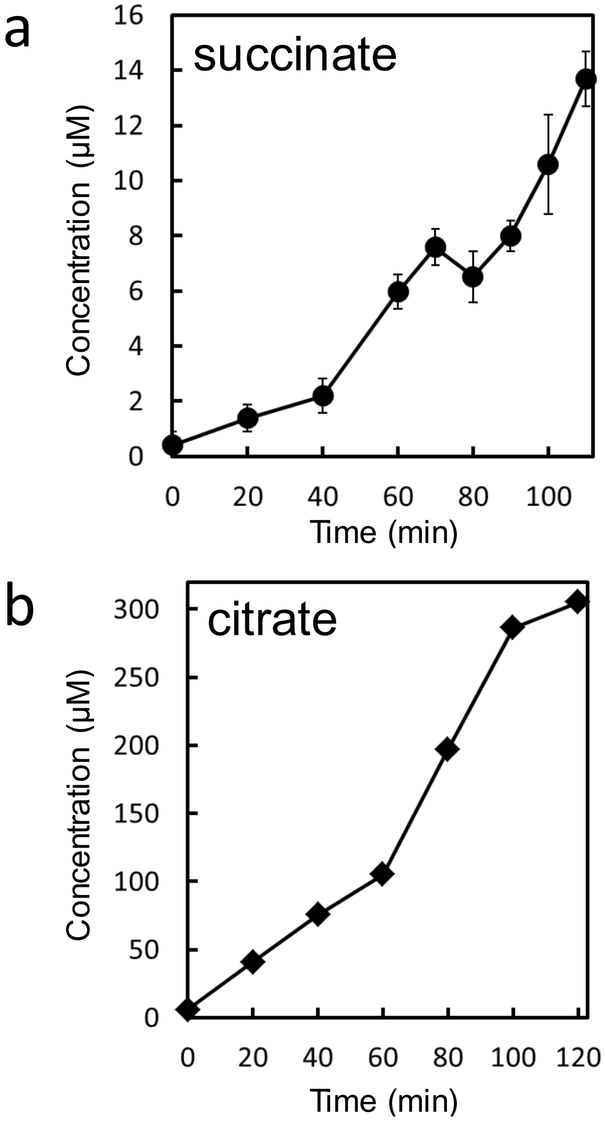

Supplementary Figure 3: Concentration of organic acids in isolated *S. tuberosum* mitochondria. a, Concentration of succinate in the mitochondria incubated with 10 mM [3-^{13}C]pyruvate, 10 mM malate and 10 mM malonate. Means and s.e.m. of duplicated experiments are shown. ; b, Concentration of citrate in the mitochondria incubated with 10 mM [3-^{13}C]glutamate, 10 mM pyruvate and 0.5 µM fluorocitrate (n=1).

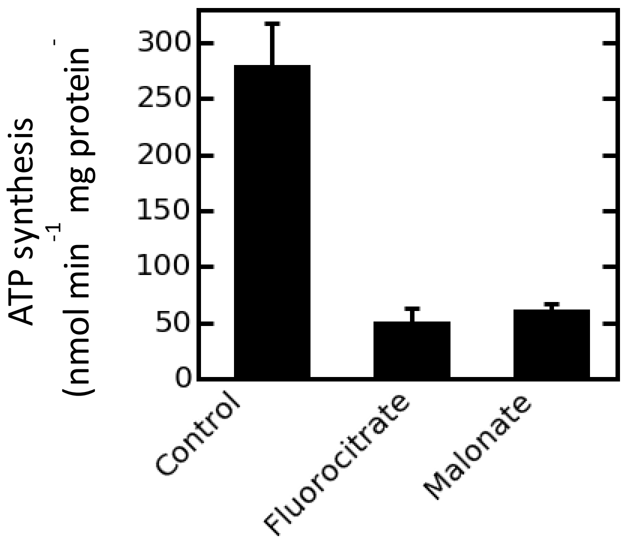

Supplementary Figure 4: The rate of ATP production during the first hour by isolated mitochondria respiring 1 mM OAA and 10 mM pyruvate plus 10 µM fluorocitrate or 10 mM malonate as indicated. Means and s.e.m. of duplicated experiments are shown. ATP synthesis was calculated from the consumption of Pi measured by ^{31}P NMR.

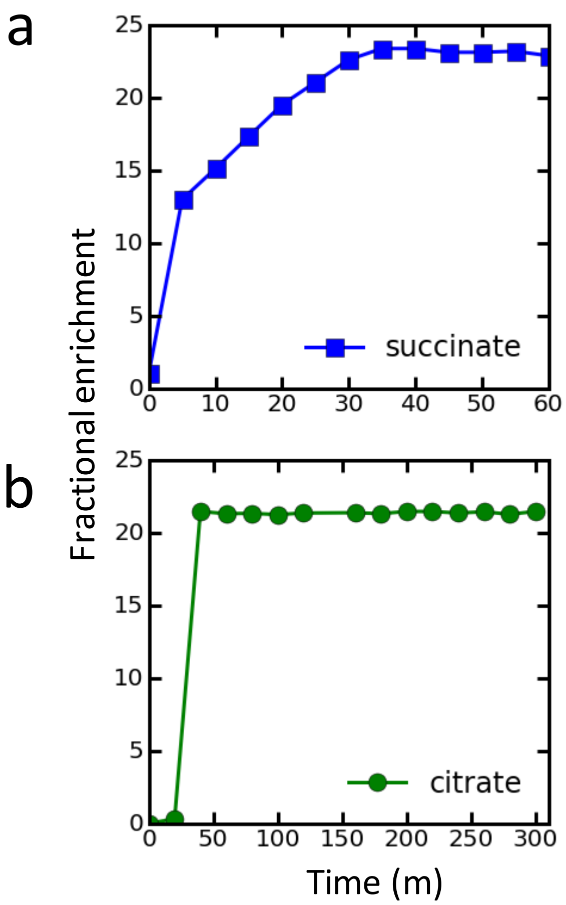

Supplementary Figure 5: Precision of measurement of labelling of succinate and citrate. a, Mitochondria were incubated with 10 mM [3-^{13}C]pyruvate, 10 mM malate and 10 mM malonate and the fractional enrichment of succinate determined over a time course (corrected for natural abundance); b, Mitochondria were incubated with 10 mM [3-^{13}C]glutamate, 10 mM pyruvate and 0.5 µM fluorocitrate and the fractional enrichment of citrate determined over a time course (corrected for natural abundance). The repeated data points once isotopic steady state had been reached (35 min for succinate, 60 min for citrate) were used to calculate the precision of the measurement. This was 0.53% for malate and 0.30% for citrate.

Supplementary Table 1: List of the proteins tested in this study

Enzyme	Subunit	Abbreviation	AGI gene code
Pyruvate dehydrogenase	E1α	PDC1a-1	At1G59900
Pyruvate dehydrogenase	E1α	PDC1a-2	At1G24180
Pyruvate dehydrogenase	E1β	PDC1b	At5G50850
Pyruvate dehydrogenase	E2	PDC2-1	At1G54220
Pyruvate dehydrogenase	E2	PDC2-2	At3G13930
Dihydrolipoamide dehydrogenase	E3	LPD1	At1g48030
Dihydrolipoamide dehydrogenase	E3	LPD2	At3g17240
Citrate synthase		CSY4	At2G44350
Aconitase		ACO2	At4G26970
Aconitase		ACO3	At2G05710
NAD^+-dependent Isocitrate dehydrogenase	Regulatory	IDH1	At4g35260
NAD^+-dependent Isocitrate dehydrogenase	Regulatory	IDH2	At2g17130
NAD^+-dependent Isocitrate dehydrogenase	Catalytic	IDH5	At5g03290
NAD^+-dependent Isocitrate dehydrogenase	Catalytic	IDH6	At3g09810
$NADP^+$-dependent Isocitrate dehydrogenase		ICDH	At5g14590
Oxoglutarate dehydrogenase	E1	ODC1-1	At3g55410
Oxoglutarate dehydrogenase	E1	ODC1-2	At5g65750
Oxoglutarate dehydrogenase	E2	ODC2-1	At4g26910
Oxoglutarate dehydrogenase	E2	ODC2-2	At5g55070
Succinyl-CoA ligase	α	SCoALa-1	At5G08300
Succinyl-CoA ligase	α	SCoALa-2	At5G23250
Succinyl-CoA ligase	β	SCoALb	At2G20420
Succinate dehydrogenase	1	SDH1-1	At5g66760
Succinate dehydrogenase	2	SDH2-1	AT3G27380
Succinate dehydrogenase	2	SDH2-2	At5g40650
Succinate dehydrogenase	2	SDH2-3	At5G65165
Succinate dehydrogenase	3	SDH3-1	At5g09600
Succinate dehydrogenase	4	SDH4	At2g46505
Succinate dehydrogenase	5	SDH5	At1g47420
Succinate dehydrogenase	6	SDH6	At1g08480
Succinate dehydrogenase	7	SDH7a	At3g47833
Succinate dehydrogenase	7	SDH7b	At5g62575
Succinate dehydrogenase	8	SDH8	At2g46390
Fumrase		FUM1	At2g47510
Malate dehydrogenase		MDH1	At1G53240
Malate dehydrogenase		MDH2	At3G15020
NAD^+-dependent malic enzyme		ME1	At2g13560
NAD^+-dependent malic enzyme		ME2	At4g00570

All constitutively expressed TCA cycle enzyme subunits and related mitochondrial enzyme proteins were tested. The enzyme names and the abbreviations are those used in the text.

Supplementary Table 2: Protein-protein interactions identified by multiple approach detection

Protein1	Protein2	AP-MS	split-LUC	Y2H
Detected by 3 techniques (11 pairs)				
ACO3	ACO2	6.84	4.4	5
IDH5	IDH2	5.41	5.35	5
PDC1a-1	LPD1	11.075	5.875	5
PDC1a-2	LPD1	6.235	6.1666667	5
PDC1a-2	ME1	4.86	5.7233333	3
PDC2-1	PDC1a-1	17.175	4.25	3
PDC2-1	PDC1b	15.065	3.925	3
PDC2-2	PDC1a-1	14.475	4.575	3
PDC2-2	PDC1b	16.12	3.775	3
SCoALa-1	ME1	5.255	4.25	5
SDH2-3	SDH1-1	15.63	5.25	5
Detected by 2 techniques (129 pairs)				
CSY4	ACO2	0.62	5.525	3
ICDH	FUM1	4.7	3.525	5
IDH1	ACO3	115.255	3.525	5
IDH2	CSY4	1.115	4.7	5
IDH2	ICDH	6.8	2.9	5
IDH2	IDH1	0.315	5.025	3
IDH5	ICDH	1.82	3.75	5
IDH6	ACO3	1.305	3.925	5
IDH6	IDH5	6.155	2.325	3
LPD1	FUM1	5.195	3.825	ND
LPD1	IDH2	0.285	5	5
LPD1	IDH6	0.205	3.7533333	5
LPD2	IDH1	5.75	3.125	5
LPD2	IDH2	1.39	5.825	5
LPD2	LPD1	4.98	2.2	3
MDH1	ACO3	74.145	2.1	5
MDH1	CSY4	3.17	3.75	5
MDH1	ICDH	4.965	3.375	5
MDH1	IDH1	8.205	3.125	5
MDH1	IDH2	6.085	2.7	5
MDH2	IDH2	0.24	3.775	3
MDH2	MDH1	879.555	2.2	5
ME1	CSY4	0.885	3.725	5
ME1	ICDH	1.605	4.2166667	5
ME1	IDH5	3.6	3.8	5
ME2	ICDH	22.33	1.525	5
ME2	LPD2	4.71	3.225	5
ME2	ME1	162.43	2.1266667	3
ODC1-1	IDH6	4.365	2.525	5
ODC1-2	ODC1-1	87.365	1.85	5
ODC2-1	CSY4	7.3	2.225	5
ODC2-1	ICDH	5.065	2.55	3
ODC2-1	IDH2	3.36	4.875	3
ODC2-1	IDH6	4.195	3.1466667	3
ODC2-1	ME1	4.62	3.3375	5
ODC2-1	ODC1-1	269.03	2.45	5
ODC2-1	ODC1-2	533.02	2.05	5
ODC2-2	ACO3	0.895	4.8	5
ODC2-2	IDH1	5.81	2.75	5
ODC2-2	LPD2	6.46	1.8	5
ODC2-2	MDH2	4.665	2.975	5
ODC2-2	ODC1-1	646.99	2.2	5
ODC2-2	ODC1-2	281.335	2.15	3

PDC1a-1	ACO2	4.635	5.625	ND
PDC1a-1	ACO3	2.26	4.025	5
PDC1a-1	ICDH	1.28	4.725	5
PDC1a-1	LPD2	0.85	4.875	5
PDC1a-1	ME1	1.28	5.375	5
PDC1a-2	ACO3	1.53	4.2	5
PDC1a-2	CSY4	6.505	3.025	5
PDC1a-2	IDH5	3.07	3.875	5
PDC1a-2	IDH6	8.87	2.35	5
PDC1a-2	LPD2	1.105	3.9	5
PDC1a-2	ME2	4.605	2.725	5
PDC1a-2	ODC2-2	2.44	4.9333333	5
PDC1a-2	PDC1a-1	17.025	4.175	8
PDC1b	IDH2	1.055	4.25	5
PDC1b	LPD1	12.075	3.775	ND
PDC1b	LPD2	3.71	4.5	5
PDC1b	ME1	0.895	5.025	3
PDC1b	PDC1a-1	46.705	3.275	5
PDC1b	PDC1a-2	31.1	3.975	8
PDC2-1	ME1	1.46	4	3
PDC2-1	ODC2-1	6.34	3.2	5
PDC2-1	PDC1a-2	10.01	2.225	5
PDC2-2	LPD1	32.925	3.3	3
PDC2-2	MDH2	10.805	2.775	5
PDC2-2	ME1	5.17	4.6	ND
PDC2-2	ODC2-2	6.105	2.675	5
PDC2-2	PDC1a-2	31.535	2.225	5
PDC2-2	PDC2-1	18.9	2.45	5
SCoALa-1	IDH2	4.665	2.425	5
SCoALa-1	LPD2	11.665	2.375	5
SCoALa-1	PDC1a-1	0.745	4.275	5
SCoALa-2	LPD2	3.91	2.575	5
SCoALa-2	ODC2-1	9.64	2.675	5
SCoALa-2	SCoALa-1	170.425	2.025	3
SCoALb	IDH2	1.865	5.45	5
SCoALb	ME1	7.01	3.75	8
SCoALb	SCoALa-1	50.935	3.225	3
SCoALb	SCoALa-2	55.725	3.25	3
SDH1-1	LPD2	9.46	2.4	5
SDH2-1	CSY4	121.19	2.475	5
SDH2-1	FUM1	4.595	2.25	5
SDH2-1	SDH1-1	15.93	3.35	5
SDH2-2	IDH2	0.39	3.82	5
SDH2-2	IDH5	0.82	3.85	5
SDH2-2	PDC1b	10.76	2.85	5
SDH2-2	PDC2-1	0.98	4.025	5
SDH2-2	SDH1-1	18.67	2.25	5
SDH2-2	SDH2-1	3.955	2.7	5
SDH2-3	LPD1	5.015	3.825	ND
SDH2-3	MDH2	0.09	4.5	5
SDH2-3	ODC1-1	2.48	4.95	3
SDH2-3	SDH2-2	0.26	4.125	5
SDH3-1	LPD2	1.405	4.225	5
SDH3-1	ODC2-2	20.31	3.3	3
SDH3-1	SDH2-2	5.72	2.925	5
SDH4	IDH2	1.13	5.1533333	5
SDH4	LPD1	16.885	1.925	5
SDH4	LPD2	6.28	1.9825	5
SDH4	MDH1	4.55	2.2	5
SDH4	ME2	5.08	3.9833333	ND
SDH4	PDC1a-2	7.94	4.3933333	ND
SDH4	PDC2-1	6.705	3.1	3

SDH4	SDH2-3	2.21	7.3575	5
SDH5	MDH2	2.4	4.675	3
SDH5	ODC1-1	5.22	2.15	5
SDH5	ODC2-1	3.9	4.125	5
SDH5	PDC1a-2	4.32	2	5
SDH5	SDH1-1	16.37	2.325	5
SDH5	SDH2-3	3.16	3.95	5
SDH6	MDH1	9.72	2.98	5
SDH6	MDH2	0	4.675	3
SDH6	SDH2-3	3.31	4.65	5
SDH6	SDH3-1	0	4	5
SDH7a	ME1	1.67	4.38	5
SDH7a	PDC2-1	0.77	4.675	5
SDH7a	SDH2-1	5.99	2.225	5
SDH7a	SDH5	1.455	4	5
SDH7b	MDH2	0	3.875	5
SDH7b	PDC1a-1	1.895	4.225	5
SDH7b	PDC2-1	1.56	4.3	5
SDH7b	SDH1-1	8.845	1.975	5
SDH7b	SDH5	1.79	3.75	5
SDH8	PDC1a-1	9.22	4.45	8
SDH8	PDC1a-2	5.015	5.0666667	ND
SDH8	PDC2-1	2.335	4.225	5
SDH8	SDH6	0	3.775	5

The protein pairs which were detected by at least two techniquies are listed. The highest scores of reciprocal pairs in individual tests (AP-MS, split-LUC and Y2H) are shown. The scores higher than thresholds are highlighted in yellow.

Supplementary Table 3: Protein-protein interactions identified by compromise based detection

Protein1	Protein2	Score
CSY4	ACO2	1
MDH2	MDH1	0.966511339
PDC1b	ME1	0.963688684
IDH2	IDH1	0.96339327
SDH2-3	ODC1-1	0.958396697
ODC2-1	IDH2	0.954008778
SDH5	MDH2	0.938939264
PDC2-2	PDC1a-1	0.937799198
SDH6	MDH2	0.937716862
PDC2-1	PDC1a-1	0.915481001
PDC1a-2	ME1	0.912306919
ODC2-2	ODC1-2	0.896930902
SDH4	SDH2-3	0.895645585
PDC2-1	PDC1b	0.890712907
PDC2-1	ME1	0.889251124
PDC2-2	PDC1b	0.88031484
MDH2	IDH2	0.872226614
SCoALb	SCoALa-2	0.862213067
LPD2	ACO3	0.861866747
SDH2-3	LPD2	0.858071355
SCoALb	SCoALa-1	0.857950787
IDH2	ACO2	0.857885448
PDC2-2	LPD1	0.854245387
PDC2-1	LPD1	0.850656291
ODC2-2	ODC1-1	0.848058043
SDH3-1	ODC2-2	0.847820137
SCoALb	PDC2-2	0.841217472
IDH1	CSY4	0.839305769
CSY4	ACO3	0.838081668
SCoALa-2	SCoALa-1	0.831327806
SDH4	PDC2-1	0.826310093
MDH2	ICDH	0.825053737
ODC2-1	IDH6	0.825031664
SDH6	SDH5	0.823778702
LPD2	ICDH	0.823564782
ME2	ME1	0.821787972
SCoALa-2	IDH2	0.821306732
MDH2	IDH6	0.819395029
SDH3-1	PDC1b	0.812183699
SDH3-1	SDH2-1	0.809200695
SDH7b	SDH7a	0.804669316
SDH7a	ODC2-2	0.802809394
SDH5	PDC1b	0.800543704
IDH1	ACO2	0.795811137
PDC1a-1	LPD1	0.792082482
SCoALa-1	ACO2	0.788607448
ODC1-1	ME1	0.7879156
ODC2-1	ICDH	0.785378264
ME2	IDH2	0.784290835
LPD2	IDH2	0.783504443
ODC2-1	ODC1-2	0.779073816
ODC2-2	IDH6	0.77547595
IDH6	IDH5	0.769530316
SCoALb	IDH6	0.768577014
PDC2-1	ME2	0.763799454
LPD2	LPD1	0.759819002
SDH6	ODC2-1	0.759105088
SCoALb	IDH2	0.75640784

MDH2	FUM1	0.753935341
IDH5	IDH2	0.750923153
PDC1a-1	ME1	0.750642172
SDH2-3	SDH1-1	0.744857825
LPD1	LPD1	0.744524535
SDH3-1	IDH6	0.73594547
ME1	LPD1	0.735752943
SCoALa-1	SCoALa-1	0.735411689
MDH2	MDH2	0.729943981
ODC2-2	ODC2-1	0.72826742
LPD1	IDH2	0.722796847
ODC2-1	ODC2-1	0.720831136
SDH8	SDH2-2	0.720831136
IDH6	ICDH	0.718803133
PDC1a-1	LPD2	0.713971775
ODC2-2	LPD1	0.713726766
PDC1a-1	ICDH	0.703255374
IDH2	CSY4	0.701348765
SDH7a	PDC2-1	0.699350476
SDH6	SDH2-3	0.698821615
PDC1a-2	LPD1	0.698488849
SCoALb	SCoALb	0.69167003
PDC1b	LPD2	0.688089934
SDH2-3	MDH2	0.686246145
ACO3	ACO2	0.682393874
SDH7a	ME1	0.678302561
IDH1	ACO3	0.673823415
ODC2-1	ODC1-1	0.673775801
SDH7b	PDC2-1	0.672414313
SCoALa-1	ME1	0.670651164
SCoALa-1	PDC1a-1	0.670176636
PDC1b	IDH2	0.668511961
SDH8	PDC2-1	0.667341339
SDH7b	PDC1a-1	0.667117232
SDH3-1	LPD2	0.666867659
PDC1a-2	ACO3	0.665108756
SDH5	ODC2-1	0.660848171
SDH2-3	SDH2-2	0.658994194
PDC1a-1	ACO3	0.652722586
SDH2-2	PDC2-1	0.652070638
SDH7a	SDH5	0.650490003
SDH6	SDH3-1	0.649748922
SDH5	SDH2-3	0.64771328
IDH6	ACO3	0.644945896
PDC1a-2	IDH5	0.642199732
SDH7b	MDH2	0.640636076
SDH4	IDH2	0.640482596
SDH2-2	IDH5	0.639177681
ME1	IDH5	0.637001972
SDH8	SDH6	0.6333458
MDH1	CSY4	0.63313782
IDH5	ICDH	0.632450219
SDH7b	SDH5	0.632434939
ME1	CSY4	0.630151422
PDC1a-2	ODC2-2	0.629120868
SCoALa-2	PDC1a-1	0.628148039
SDH7a	SDH2-3	0.628010519
SDH8	IDH1	0.62605807
SDH6	SDH4	0.625217412
SDH4	SCoALb	0.624464701
ODC2-2	IDH5	0.623131093
SDH2-1	ME2	0.622985933

SDH2-2	SCoALb	0.621374737
SDH3-1	IDH5	0.621290697
ODC2-2	ACO3	0.62104367
SDH5	IDH1	0.62101311
IDH2	ACO3	0.620992736
SDH7b	SDH2-3	0.62087559
LPD2	CSY4	0.620794096
PDC1b	PDC1a-1	0.620682868
PDC1a-2	MDH2	0.619666768
SDH8	ME2	0.619531795
SDH1-1	PDC1b	0.619310234
SDH2-3	PDC2-1	0.619177807
ICDH	FUM1	0.617513979
ODC1-1	MDH2	0.615438442
SDH7b	CSY4	0.615145575
SDH6	SDH6	0.613297539
SDH1-1	CSY4	0.612012318
SDH2-1	SDH1-1	0.610475817
ODC1-1	ACO3	0.610350189
SDH2-1	LPD2	0.610075148
SDH8	SDH1-1	0.609278888
SCoALb	LPD2	0.608823033
MDH2	CSY4	0.607997912
SDH2-1	IDH2	0.607759882
SDH4	CSY4	0.607344181
MDH1	ICDH	0.606713538
SDH8	PDC2-2	0.60663629
ACO3	ACO3	0.606575681
PDC1b	ODC1-1	0.604706761
SDH2-2	IDH2	0.604383334
SDH7a	SDH3-1	0.604184694
PDC1b	ME2	0.604177901
ODC2-1	ME1	0.603803964
ME2	MDH2	0.602973325
SDH4	SDH2-1	0.602482581
LPD1	IDH5	0.600598129
SDH7b	IDH1	0.600549742
SDH2-1	CSY4	0.60029841

The protein pairs with compromise score above 0.6 are listed. The compromise scores of each pair are shown.

Supplementary Table 4: Amino acid sequence similarity between TCA cycle enzymes of *Arabidopsis thaliana* and *Solanum tuberosum*

Enzyme	Abbreviation	AGI code	Potato Accession	Identity	Positive	Score	E value	Coverage
Pyruvate dehydrogenase	PDC1a-1	At1G59900	Sotub05g011910.1.1	81%	89%	1661	0.00E+00	96%
Pyruvate dehydrogenase	PDC1a-2	At1G24180	Sotub05g011910.1.1	76%	88%	1599	0.00E+00	96%
Pyruvate dehydrogenase	PDC1b	At5G50850	Sotub06g028390.1.1	87%	93%	1604	0.00E+00	94%
Pyruvate dehydrogenase	PDC2-1	At1G54220	Sotub07g007990.1.1	65%	77%	1838	0.00E+00	100%
Pyruvate dehydrogenase	PDC2-2	At3G13930	Sotub07g007990.1.1	68%	78%	1876	0.00E+00	100%
Dihydrolipoamide dehydrogenase	LPD1	At1g48030	Sotub05g025970.1.1	83%	92%	2180	0.00E+00	100%
Dihydrolipoamide dehydrogenase	LPD2	At3g17240	Sotub12g031040.1.1	83%	91%	2158	0.00E+00	100%
Citrate synthase	CSY4	At2G44350	Sotub01g027350.1.1	82%	91%	2094	0.00E+00	99%
Aconitase	ACO2	At4G26970	Sotub07g019480.1.1	78%	87%	4193	0.00E+00	98%
Aconitase	ACO3	At2G05710	Sotub12g011890.1.1	87%	93%	4471	0.00E+00	95%
NAD^+-dependent Isocitrate dehydrogenase	IDH1	At4g35260	Sotub10g017390.1.1	83%	90%	1655	0.00E+00	100%
NAD^+-dependent Isocitrate dehydrogenase	IDH2	At2g17130	Sotub10g017390.1.1	79%	88%	1589	0.00E+00	100%
NAD^+-dependent Isocitrate dehydrogenase	IDH5	At5g03290	Sotub02g006980.1.1	83%	89%	1651	0.00E+00	99%
NAD^+-dependent Isocitrate dehydrogenase	IDH6	At3g09810	Sotub08g023480.1.1	78%	88%	1567	0.00E+00	99%
$NADP^+$-dependent Isocitrate dehydrogenase	ICDH	At5g14590	Sotub02g036290.1.1	82%	92%	1989	0.00E+00	89%
Oxoglutarate dehydrogenase	ODC1-1	At3g55410	Sotub04g010270.1.1	85%	93%	4859	0.00E+00	100%
Oxoglutarate dehydrogenase	ODC1-2	At5g65750	Sotub05g027690.1.1	84%	92%	4808	0.00E+00	100%
Oxoglutarate dehydrogenase	ODC2-1	At4g26910	Sotub07g029040.1.1	70%	80%	1622	0.00E+00	100%
Oxoglutarate dehydrogenase	ODC2-2	At5g55070	Sotub07g029040.1.1	69%	79%	1603	0.00E+00	100%
Succinyl-CoA ligase	SCoALa-1	At5G08300	Sotub01g008180.1.1	82%	88%	1472	0.00E+00	99%
Succinyl-CoA ligase	SCoALa-2	At5G23250	Sotub01g008180.1.1	84%	90%	1486	0.00E+00	100%
Succinyl-CoA ligase	SCoALb	At2G20420	Sotub06g034240.1.1	87%	95%	1962	0.00E+00	100%
Succinate dehydrogenase	SDH1-1	At5g66760	Sotub02g028490.1.1	90%	95%	3044	0.00E+00	100%
Succinate dehydrogenase	SDH2-1	AT3G27380	Sotub02g036880.1.1	78%	87%	1158	1.00E-162	100%
Succinate dehydrogenase	SDH2-2	At5g40650	Sotub02g036880.1.1	79%	87%	1168	1.00E-163	99%
Succinate dehydrogenase	SDH2-3	At5G65165	Sotub04g020790.1.1	74%	83%	1098	1.00E-152	90%
Succinate dehydrogenase	SDH3-1	At5g09600	Sotub03g018920.1.1	46%	63%	212	6.00E-22	41%
Succinate dehydrogenase	SDH4	At2g46505	Sotub09g006140.1.1	56%	82%	154	5.00E-14	33%
Succinate dehydrogenase	SDH5	At1g47420	Sotub04g032250.1.1	72%	83%	725	6.00E-97	72%
Succinate dehydrogenase	SDH6	At1g08480	Sotub11g028240.1.1	70%	80%	537	1.00E-71	98%
Succinate dehydrogenase	SDH7a	At3g47833	Sotub06g027620.1.1	69%	80%	324	9.00E-41	98%
Succinate dehydrogenase	SDH7b	At5g62575	Sotub06g027620.1.1	68%	83%	337	1.00E-42	93%
Succinate dehydrogenase	SDH8	At2g46390	Sotub01g034520.1.1	57%	69%	86	6.00E-06	70%
Fumrase	FUM1	At2g47510	Sotub09g022990.1.1	85%	93%	2250	0.00E+00	100%
Malate dehydrogenase	MDH1	At1G53240	Sotub07g024580.1.1	82%	92%	1516	0.00E+00	100%
Malate dehydrogenase	MDH2	At3G15020	Sotub07g024580.1.1	82%	91%	1485	0.00E+00	100%
NAD^+-dependent malic enzyme	ME1	At2g13560	Sotub08g008850.1.1	83%	92%	2772	0.00E+00	100%
NAD^+-dependent malic enzyme	ME2	At4g00570	Sotub01g031560.1.1	77%	88%	2543	0.00E+00	100%

The amino acid sequences of Arabidopsis TCA cycle proteins were searched against ITAG *Solanum tuberosum* group *Phureja* DM1-3 CDS (v1) database by blastp search tool in the Spud DB database (http://solanaceae.plantbiology.msu.edu/integrated_searches.shtml, accessed on 10th Oct 2016). The *S. tuberosum* CDS showed the highest Score in blastp search are shown.

Supplementary Table 5: Primers used for cDNA cloning*

AGI code	Abbreviation	5' primer for the first nested PCR**	3' primer for the first nested PCR**	5' primer	3' primer
At1G59900	PDC1a-1			ATGGCTCTATCACGCCTCTCATC	TGGAAGGGAAGCTTTGACTTCTTTT
At1G24180	PDC1a-2			ATGGCGTTATCACGACTTTCTTC	TGGAAGTGTTACTTTGAGTTCTTTTCTG
At5G50850	PDC1b			ATGTTGGGAATCTTGAGGCA	TTTCGATCTGTAACAAGCTCTCTTT
At1G54220	PDC2-1			ATGGCTTATGCGTCACGC	GAGTAACATAGATTTAGGGTTCTCGATG
At3G13930	PDC2-2			ATGGCTTCTCGTATCATCAATCATT	AGCAACATAGATTCTGGGGTCTC
At1g48030	LPD1	(Primer for BP reaction)	GCTAAACCACAGAAAACCGAACCAA	ATGGCGATGGCGAGTTTAGCTAG	GATGTGAATAGGCTTGTCATAGGTGGC
At3g17240	LPD2	ATTCTTTCGTGACTTGCTCAAAGCAG	TACCCCATAATGAACAATGCAACTCC	ATGGCGATGGCGAGCTTAG	CATGTGAATGGGCTTGTCATAGG
At2G44350	CSY4			AGAATGGTGTTTTTCCGCAG	AGCAGATGAAGCTTTCTTACAATG
At4G26970	ACO2			ATGTATCGACGCGCCACTTCC	CTTGGCGCTCAAACTCCGG
At2G05710	ACO3			ATGTATTTAACCGCTTCATCTTCCGC	TTGCTTGCTCAAGTTTCTGATAACATATGG
At4g35260	IDH1			ATGTCTCGCAGATCGCTAACTCTCC	GTCTAGTTTTGCAATGACCGCATCA
At2g17130	IDH2			ATGTCTCGCCAATCGTTTTCTCTACTG	ATCCAAATTTGCAATGACAGCGTC
At5g03290	IDH5			ATGACCATGGCAGCAAATCTA	GAGATGATCACAGATTGCCTTTG
At3g09810	IDH6			ATGACCATGACAGCATTTCTCGC	GAGATGATCACAGATGGCTTTTGTGA
At5g14590	ICDH			ATGCTGAATAAGCTCACGCACG	GACAAGAGGAAGCTCTTTGAACTGTGT
At3g55410	ODC1-1			ATGGTGTGGTTTCGTGCTGGTTC	ATTGATGGGTTCCTTTCCGATGG
At5g65750	ODC1-2			ATGGTTTGGTTTAGAATCGGTTCTT	AGGGATGACGGGGGTGAT
At4g26910	ODC2-1	CTCATATGCGTTTACCGATCTTCTCG	CAAAAGCCAGACACTTTGTGACTCCT	ATGATGATGCGTGCTGTTATAAGGA	TATGTCAAGAAGAAGCCTTTGTGGAT
At5g55070	ODC2-2	ATTCTTTCGTGACTTGCTCAAAGCAG	TACCCCATAATGAACAATGCAACTCC	ATGATGTTGCGTGCTGTTTTTAGGA	TATGTCGAGAAGAAGCCTCTGAGGATC
At5G08300	SCoALa-1			ATGTCTAGACAAGTGGCAAGGCTTA	CTGCTTCAAAAGACCTCTTTCTTGA
At5G23250	SCoALa-2			ATGTCTAGGCAGGTCACGAGACTT	CTTCAAAAGACCTCTTTCTTGGAAAAG
At2G20420	SCoALb			ATGAGGGGATTGGTGAACAA	GTGAGCTAAAGCTTTAACCGCT
At5g66760	SDH1-1			ATGTGGCGCTGCGTCTCT	ATAGACACGAGCTTTGGGAGGG
At3G27380	SDH2-1			ATGGCGTCTGGTTTGATCG	ACGCTGAAGTTGCTTGATGTG
At5g40650	SDH2-2	ACAATTTCATTTCATTTGCTCTCTTCTCC	AGGCAAAGCTGAATGAGTGGATGATA	ATGGCGTTTGGTTTGATCGG	ACCAGATTTCTGAAGCTGCTTGATGT
At5G65165	SDH2-3			ATGTCGTCTGTCTTGCGGTTGTT	GACACTCTCTGTTCTCACTAACGGATCG
At5g09600	SDH3-1			ATGGCTGCCACGGCTCTTT	ATGGGTCAAGAGTGAGTGAGTACT

At2g46505	SDH4	ACATAGGGTTTGTTTCTCCGATCA	CAACACACAACAAAAGCTTGAAGG	ATGTCTCTCCGCCGCACT	CTTGA<br>GAGAAGAAACAAGATAATATCTTT<br>AAGAA
At1g47420	SDH5			ATGGGAACCCTAGGACGAGCG	GGCCCTTTGCTCCACGTAAGATC
At1g08480	SDH6			ATGGGAGATTCGGAGTCATTTGTTG	TTGAGACTTACGTTCCCACCATTC<br>C
At3g47833	SDH7a			ATGGCGTTTTTACTCAACACCTCG	TGCAGCCTGAGCTTCCTTTCTC
At5g62575	SDH7b			ATGGCGTTTTTGCTCAACAACG	GAGCTTTTCCCTGCAGCCA
At2g46390	SDH8			ATGATTTACCGAAAGTGGAGTTTGC	CTTGTTTGTTGAGACATTCTTCTT<br>CTGC
At2g47510	FUM1			ATGTCGATTTACGTCGCGT	ATCGGAGGGACCAATCATC
At1G53240	MDH1	CGATTGCTTCAGATTCGTCA	GGAGGCACAAACATGGATTT	ATGTTCAGATCTATGCTCGTC	CTGGTTGGCAAACTTGACTC
At3G15020	MDH2			ATGTTCCGATCAATGATTGTTCGAT	TTGGTTGGCAAATTTGATGCC
At2g13560	ME1			ATGGGAATAGCCAATAAGCTCC	GTCATCCTTGTAGACCAAAGTCG
At4g00570	ME2			ATGATGTGGAAGAACATTGCTGGG	TTTCTCGTGAACGAGAGGGCTGTA

*, adapter sequences (GGGGACAAGTTTGTACAAAAAAGCAGGCTCCACC– and GGGGACCACTTTGTACAAGAAAGCTGGGTC– for 5’ and 3’ primers, respectively) were attached to the 5' end of each primer for Gateway BP recombination reaction.

**, some genes which have very similar sequences to other genes at the ends of coding sequence were amplified first by primer sets annealing to untranslated regions and then amplified by primers for the amplification of coding sequence using the first PCR products as template. The primers used for the first PCR are shown here.

Supplementary Table 6: Plasmids constructed in this study

AGI code	Abbreviation	Donor vector	Entry vector	Destination vector	Expression vector	Purpose
At1G59900	PDC1a-1	pDONR207	pENTR_PDC1a-1	pK7FWG2	pKF_PDC1a-1	C-term GFP fusion for AP-MS
At1G59900	PDC1a-1	pDONR207	pENTR_PDC1a-1	pDuExAc6	pDuExAc_PDC1a-1	C-term N-Luc fusion for split-LUC
At1G59900	PDC1a-1	pDONR207	pENTR_PDC1a-1	pDuExDc6	pDuExDc_PDC1a-1	C-term C-Luc fusion for split-LUC
At1G59900	PDC1a-1	pDONR207	pENTR_PDC1a-1	pGBKCg	pGBKCg_PDC1a-1	C-term DBD fusion for Y2H
At1G59900	PDC1a-1	pDONR207	pENTR_PDC1a-1	pGADCg	pGADCg_PDC1a-1	C-term AD fusion for Y2H
At1G59900	PDC1a-1	pDONR207	pENTR_PDC1a-1	pDuScyCE	pDuScyCE_PDC1a-1	C-term C-SCF fusion for BiFC
At1G24180	PDC1a-2	pDONR207	pENTR_PDC1a-2	pK7FWG2	pKF_PDC1a-2	C-term GFP fusion for AP-MS
At1G24180	PDC1a-2	pDONR207	pENTR_PDC1a-2	pDuExAc6	pDuExAc_PDC1a-2	C-term N-Luc fusion for split-LUC
At1G24180	PDC1a-2	pDONR207	pENTR_PDC1a-2	pDuExDc6	pDuExDc_PDC1a-2	C-term C-Luc fusion for split-LUC
At1G24180	PDC1a-2	pDONR207	pENTR_PDC1a-2	pGBKCg	pGBKCg_PDC1a-2	C-term DBD fusion for Y2H
At1G24180	PDC1a-2	pDONR207	pENTR_PDC1a-2	pGADCg	pGADCg_PDC1a-2	C-term AD fusion for Y2H
At1G24180	PDC1a-2	pDONR207	pENTR_PDC1a-2	pDuScyCE	pDuScyCE_PDC1a-2	C-term C-SCF fusion for BiFC
At5G50850	PDC1b	pDONR207	pENTR_PDC1b	pK7FWG2	pKF_PDC1b	C-term GFP fusion for AP-MS
At5G50850	PDC1b	pDONR207	pENTR_PDC1b	pDuExAc6	pDuExAc_PDC1b	C-term N-Luc fusion for split-LUC
At5G50850	PDC1b	pDONR207	pENTR_PDC1b	pDuExDc6	pDuExDc_PDC1b	C-term C-Luc fusion for split-LUC
At5G50850	PDC1b	pDONR207	pENTR_PDC1b	pGBKCg	pGBKCg_PDC1b	C-term DBD fusion for Y2H
At5G50850	PDC1b	pDONR207	pENTR_PDC1b	pGADCg	pGADCg_PDC1b	C-term AD fusion for Y2H
At5G50850	PDC1b	pDONR207	pENTR_PDC1b	pDuScyCE	pDuScyCE_PDC1b	C-term C-SCF fusion for BiFC
At1G54220	PDC2-1	pDONR207	pENTR_PDC2-1	pK7FWG2	pKF_PDC2-1	C-term GFP fusion for AP-MS
At1G54220	PDC2-1	pDONR207	pENTR_PDC2-1	pDuExAc6	pDuExAc_PDC2-1	C-term N-Luc fusion for split-LUC
At1G54220	PDC2-1	pDONR207	pENTR_PDC2-1	pDuExDc6	pDuExDc_PDC2-1	C-term C-Luc fusion for split-LUC
At1G54220	PDC2-1	pDONR207	pENTR_PDC2-1	pGBKCg	pGBKCg_PDC2-1	C-term DBD fusion for Y2H
At1G54220	PDC2-1	pDONR207	pENTR_PDC2-1	pGADCg	pGADCg_PDC2-1	C-term AD fusion for Y2H
At3G13930	PDC2-2	pDONR207	pENTR_PDC2-2	pK7FWG2	pKF_PDC2-2	C-term GFP fusion for AP-MS
At3G13930	PDC2-2	pDONR207	pENTR_PDC2-2	pDuExAc6	pDuExAc_PDC2-2	C-term N-Luc fusion for split-LUC
At3G13930	PDC2-2	pDONR207	pENTR_PDC2-2	pDuExDc6	pDuExDc_PDC2-2	C-term C-Luc fusion for split-LUC
At3G13930	PDC2-2	pDONR207	pENTR_PDC2-2	pGBKCg	pGBKCg_PDC2-2	C-term DBD fusion for Y2H
At3G13930	PDC2-2	pDONR207	pENTR_PDC2-2	pGADCg	pGADCg_PDC2-2	C-term AD fusion for Y2H
At1g48030	LPD1	pDONR207	pENTR_LPD1	pK7FWG2	pKF_LPD1	C-term GFP fusion for AP-MS
At1g48030	LPD1	pDONR207	pENTR_LPD1	pDuExAc6	pDuExAc_LPD1	C-term N-Luc fusion for split-LUC
At1g48030	LPD1	pDONR207	pENTR_LPD1	pDuExDc6	pDuExDc_LPD1	C-term C-Luc fusion for split-LUC
At1g48030	LPD1	pDONR207	pENTR_LPD1	pGBKCg	pGBKCg_LPD1	C-term DBD fusion for Y2H
At1g48030	LPD1	pDONR207	pENTR_LPD1	pGADCg	pGADCg_LPD1	C-term AD fusion for Y2H
At3g17240	LPD2	pDONR207	pENTR_LPD2	pK7FWG2	pKF_LPD2	C-term GFP fusion for AP-MS
At3g17240	LPD2	pDONR207	pENTR_LPD2	pDuExAc6	pDuExAc_LPD2	C-term N-Luc fusion for split-LUC
At3g17240	LPD2	pDONR207	pENTR_LPD2	pDuExDc6	pDuExDc_LPD2	C-term C-Luc fusion for split-LUC
At3g17240	LPD2	pDONR207	pENTR_LPD2	pGBKCg	pGBKCg_LPD2	C-term DBD fusion for Y2H
At3g17240	LPD2	pDONR207	pENTR_LPD2	pGADCg	pGADCg_LPD2	C-term AD fusion for Y2H
At2G44350	CSY4	pDONR207	pENTR_CSY4	pK7FWG2	pKF_CSY4	C-term GFP fusion for AP-MS
At2G44350	CSY4	pDONR207	pENTR_CSY4	pDuExAc6	pDuExAc_CSY4	C-term N-Luc fusion for split-LUC

At2G44350	CSY4	pDONR207	pENTR_CSY4	pDuExDc6	pDuExDc_CSY4	C-term C-Luc fusion for split-LUC
At2G44350	CSY4	pDONR207	pENTR_CSY4	pGBKCg	pGBKCg_CSY4	C-term DBD fusion for Y2H
At2G44350	CSY4	pDONR207	pENTR_CSY4	pGADCg	pGADCg_CSY4	C-term AD fusion for Y2H
At2G44350	CSY4	pDONR207	pENTR_CSY4	pDuScyCE	pDuScyCE_CSY4	C-term C-SCF fusion for BiFC
At4G26970	ACO2	pDONR207	pENTR_ACO2	pK7FWG2	pKF_ACO2	C-term GFP fusion for AP-MS
At4G26970	ACO2	pDONR207	pENTR_ACO2	pDuExAc6	pDuExAc_ACO2	C-term N-Luc fusion for split-LUC
At4G26970	ACO2	pDONR207	pENTR_ACO2	pDuExDc6	pDuExDc_ACO2	C-term C-Luc fusion for split-LUC
At4G26970	ACO2	pDONR207	pENTR_ACO2	pGBKCg	pGBKCg_ACO2	C-term DBD fusion for Y2H
At4G26970	ACO2	pDONR207	pENTR_ACO2	pGADCg	pGADCg_ACO2	C-term AD fusion for Y2H
At4G26970	ACO2	pDONR207	pENTR_ACO2	pDuVyNE	pDuVyNE_ACO2	C-term N-Venus fusion for BiFC
At2G05710	ACO3	pDONR207	pENTR_ACO3	pK7FWG2	pKF_ACO3	C-term GFP fusion for AP-MS
At2G05710	ACO3	pDONR207	pENTR_ACO3	pDuExAc6	pDuExAc_ACO3	C-term N-Luc fusion for split-LUC
At2G05710	ACO3	pDONR207	pENTR_ACO3	pDuExDc6	pDuExDc_ACO3	C-term C-Luc fusion for split-LUC
At2G05710	ACO3	pDONR207	pENTR_ACO3	pGBKCg	pGBKCg_ACO3	C-term DBD fusion for Y2H
At2G05710	ACO3	pDONR207	pENTR_ACO3	pGADCg	pGADCg_ACO3	C-term AD fusion for Y2H
At2G05710	ACO3	pDONR207	pENTR_ACO3	pDuVyNE	pDuVyNE_ACO3	C-term N-Venus fusion for BiFC
At4g35260	IDH1	pDONR207	pENTR_IDH1	pK7FWG2	pKF_IDH1	C-term GFP fusion for AP-MS
At4g35260	IDH1	pDONR207	pENTR_IDH1	pDuExAc6	pDuExAc_IDH1	C-term N-Luc fusion for split-LUC
At4g35260	IDH1	pDONR207	pENTR_IDH1	pDuExDc6	pDuExDc_IDH1	C-term C-Luc fusion for split-LUC
At4g35260	IDH1	pDONR207	pENTR_IDH1	pGBKCg	pGBKCg_IDH1	C-term DBD fusion for Y2H
At4g35260	IDH1	pDONR207	pENTR_IDH1	pGADCg	pGADCg_IDH1	C-term AD fusion for Y2H
At2g17130	IDH2	pDONR207	pENTR_IDH2	pK7FWG2	pKF_IDH2	C-term GFP fusion for AP-MS
At2g17130	IDH2	pDONR207	pENTR_IDH2	pDuExAc6	pDuExAc_IDH2	C-term N-Luc fusion for split-LUC
At2g17130	IDH2	pDONR207	pENTR_IDH2	pDuExDc6	pDuExDc_IDH2	C-term C-Luc fusion for split-LUC
At2g17130	IDH2	pDONR207	pENTR_IDH2	pGBKCg	pGBKCg_IDH2	C-term DBD fusion for Y2H
At2g17130	IDH2	pDONR207	pENTR_IDH2	pGADCg	pGADCg_IDH2	C-term AD fusion for Y2H
At5g03290	IDH5	pDONR207	pENTR_IDH5	pK7FWG2	pKF_IDH5	C-term GFP fusion for AP-MS
At5g03290	IDH5	pDONR207	pENTR_IDH5	pDuExAc6	pDuExAc_IDH5	C-term N-Luc fusion for split-LUC
At5g03290	IDH5	pDONR207	pENTR_IDH5	pDuExDc6	pDuExDc_IDH5	C-term C-Luc fusion for split-LUC
At5g03290	IDH5	pDONR207	pENTR_IDH5	pGBKCg	pGBKCg_IDH5	C-term DBD fusion for Y2H
At5g03290	IDH5	pDONR207	pENTR_IDH5	pGADCg	pGADCg_IDH5	C-term AD fusion for Y2H
At3g09810	IDH6	pDONR207	pENTR_IDH6	pK7FWG2	pKF_IDH6	C-term GFP fusion for AP-MS
At3g09810	IDH6	pDONR207	pENTR_IDH6	pDuExAc6	pDuExAc_IDH6	C-term N-Luc fusion for split-LUC
At3g09810	IDH6	pDONR207	pENTR_IDH6	pDuExDc6	pDuExDc_IDH6	C-term C-Luc fusion for split-LUC
At3g09810	IDH6	pDONR207	pENTR_IDH6	pGBKCg	pGBKCg_IDH6	C-term DBD fusion for Y2H
At3g09810	IDH6	pDONR207	pENTR_IDH6	pGADCg	pGADCg_IDH6	C-term AD fusion for Y2H
At3g09810	IDH6	pDONR207	pENTR_IDH6	pDuScyCE	pDuScyCE_IDH6	C-term C-SCF fusion for BiFC
At5g14590	ICDH	pDONR207	pENTR_ICDH	pK7FWG2	pKF_ICDH	C-term GFP fusion for AP-MS
At5g14590	ICDH	pDONR207	pENTR_ICDH	pDuExAc6	pDuExAc_ICDH	C-term N-Luc fusion for split-LUC
At5g14590	ICDH	pDONR207	pENTR_ICDH	pDuExDc6	pDuExDc_ICDH	C-term C-Luc fusion for split-LUC
At5g14590	ICDH	pDONR207	pENTR_ICDH	pGBKCg	pGBKCg_ICDH	C-term DBD fusion for Y2H
At5g14590	ICDH	pDONR207	pENTR_ICDH	pGADCg	pGADCg_ICDH	C-term AD fusion for Y2H
At3g55410	ODC1-1	pDONR207	pENTR_ODC1-1	pK7FWG2	pKF_ODC1-1	C-term GFP fusion for AP-MS
At3g55410	ODC1-1	pDONR207	pENTR_ODC1-1	pDuExAc6	pDuExAc_ODC1-1	C-term N-Luc fusion for split-LUC
At3g55410	ODC1-1	pDONR207	pENTR_ODC1-1	pDuExDc6	pDuExDc_ODC1-1	C-term C-Luc fusion for split-LUC

At3g55410	ODC1-1	pDONR207	pENTR_ODC1-1	pGBKCg	pGBKCg_ODC1-1	C-term DBD fusion for Y2H
At3g55410	ODC1-1	pDONR207	pENTR_ODC1-1	pGADCg	pGADCg_ODC1-1	C-term AD fusion for Y2H
At3g55410	ODC1-1	pDONR207	pENTR_ODC1-1	pDuVyNE	pDuVyNE_ODC1-1	C-term N-Venus fusion for BiFC
At5g65750	ODC1-2	pDONR207	pENTR_ODC1-2	pK7FWG2	pKF_ODC1-2	C-term GFP fusion for AP-MS
At5g65750	ODC1-2	pDONR207	pENTR_ODC1-2	pDuExAc6	pDuExAc_ODC1-2	C-term N-Luc fusion for split-LUC
At5g65750	ODC1-2	pDONR207	pENTR_ODC1-2	pDuExDc6	pDuExDc_ODC1-2	C-term C-Luc fusion for split-LUC
At5g65750	ODC1-2	pDONR207	pENTR_ODC1-2	pGBKCg	pGBKCg_ODC1-2	C-term DBD fusion for Y2H
At5g65750	ODC1-2	pDONR207	pENTR_ODC1-2	pGADCg	pGADCg_ODC1-2	C-term AD fusion for Y2H
At4g26910	ODC2-1	pDONR207	pENTR_ODC2-1	pK7FWG2	pKF_ODC2-1	C-term GFP fusion for AP-MS
At4g26910	ODC2-1	pDONR207	pENTR_ODC2-1	pDuExAc6	pDuExAc_ODC2-1	C-term N-Luc fusion for split-LUC
At4g26910	ODC2-1	pDONR207	pENTR_ODC2-1	pDuExDc6	pDuExDc_ODC2-1	C-term C-Luc fusion for split-LUC
At4g26910	ODC2-1	pDONR207	pENTR_ODC2-1	pGBKCg	pGBKCg_ODC2-1	C-term DBD fusion for Y2H
At4g26910	ODC2-1	pDONR207	pENTR_ODC2-1	pGADCg	pGADCg_ODC2-1	C-term AD fusion for Y2H
At5g55070	ODC2-2	pDONR207	pENTR_ODC2-2	pK7FWG2	pKF_ODC2-2	C-term GFP fusion for AP-MS
At5g55070	ODC2-2	pDONR207	pENTR_ODC2-2	pDuExAc6	pDuExAc_ODC2-2	C-term N-Luc fusion for split-LUC
At5g55070	ODC2-2	pDONR207	pENTR_ODC2-2	pDuExDc6	pDuExDc_ODC2-2	C-term C-Luc fusion for split-LUC
At5g55070	ODC2-2	pDONR207	pENTR_ODC2-2	pGBKCg	pGBKCg_ODC2-2	C-term DBD fusion for Y2H
At5g55070	ODC2-2	pDONR207	pENTR_ODC2-2	pGADCg	pGADCg_ODC2-2	C-term AD fusion for Y2H
At5G08300	SCoALa-1	pDONR207	pENTR_SCoALa-1	pK7FWG2	pKF_SCoALa-1	C-term GFP fusion for AP-MS
At5G08300	SCoALa-1	pDONR207	pENTR_SCoALa-1	pDuExAc6	pDuExAc_SCoALa-1	C-term N-Luc fusion for split-LUC
At5G08300	SCoALa-1	pDONR207	pENTR_SCoALa-1	pDuExDc6	pDuExDc_SCoALa-1	C-term C-Luc fusion for split-LUC
At5G08300	SCoALa-1	pDONR207	pENTR_SCoALa-1	pGBKCg	pGBKCg_SCoALa-1	C-term DBD fusion for Y2H
At5G08300	SCoALa-1	pDONR207	pENTR_SCoALa-1	pGADCg	pGADCg_SCoALa-1	C-term AD fusion for Y2H
At5G23250	SCoALa-2	pDONR207	pENTR_SCoALa-2	pK7FWG2	pKF_SCoALa-2	C-term GFP fusion for AP-MS
At5G23250	SCoALa-2	pDONR207	pENTR_SCoALa-2	pDuExAc6	pDuExAc_SCoALa-2	C-term N-Luc fusion for split-LUC
At5G23250	SCoALa-2	pDONR207	pENTR_SCoALa-2	pDuExDc6	pDuExDc_SCoALa-2	C-term C-Luc fusion for split-LUC
At5G23250	SCoALa-2	pDONR207	pENTR_SCoALa-2	pGBKCg	pGBKCg_SCoALa-2	C-term DBD fusion for Y2H
At5G23250	SCoALa-2	pDONR207	pENTR_SCoALa-2	pGADCg	pGADCg_SCoALa-2	C-term AD fusion for Y2H
At2G20420	SCoALb	pDONR207	pENTR_SCoALb	pK7FWG2	pKF_SCoALb	C-term GFP fusion for AP-MS
At2G20420	SCoALb	pDONR207	pENTR_SCoALb	pDuExAc6	pDuExAc_SCoALb	C-term N-Luc fusion for split-LUC
At2G20420	SCoALb	pDONR207	pENTR_SCoALb	pDuExDc6	pDuExDc_SCoALb	C-term C-Luc fusion for split-LUC
At2G20420	SCoALb	pDONR207	pENTR_SCoALb	pGBKCg	pGBKCg_SCoALb	C-term DBD fusion for Y2H
At2G20420	SCoALb	pDONR207	pENTR_SCoALb	pGADCg	pGADCg_SCoALb	C-term AD fusion for Y2H
At5g66760	SDH1-1	pDONR207	pENTR_SDH1-1	pK7FWG2	pKF_SDH1-1	C-term GFP fusion for AP-MS
At5g66760	SDH1-1	pDONR207	pENTR_SDH1-1	pDuExAc6	pDuExAc_SDH1-1	C-term N-Luc fusion for split-LUC
At5g66760	SDH1-1	pDONR207	pENTR_SDH1-1	pDuExDc6	pDuExDc_SDH1-1	C-term C-Luc fusion for split-LUC
At5g66760	SDH1-1	pDONR207	pENTR_SDH1-1	pGBKCg	pGBKCg_SDH1-1	C-term DBD fusion for Y2H
At5g66760	SDH1-1	pDONR207	pENTR_SDH1-1	pGADCg	pGADCg_SDH1-1	C-term AD fusion for Y2H
AT3G27380	SDH2-1	pDONR207	pENTR_SDH2-1	pK7FWG2	pKF_SDH2-1	C-term GFP fusion for AP-MS
AT3G27380	SDH2-1	pDONR207	pENTR_SDH2-1	pDuExAc6	pDuExAc_SDH2-1	C-term N-Luc fusion for split-LUC
AT3G27380	SDH2-1	pDONR207	pENTR_SDH2-1	pDuExDc6	pDuExDc_SDH2-1	C-term C-Luc fusion for split-LUC
AT3G27380	SDH2-1	pDONR207	pENTR_SDH2-1	pGBKCg	pGBKCg_SDH2-1	C-term DBD fusion for Y2H
AT3G27380	SDH2-1	pDONR207	pENTR_SDH2-1	pGADCg	pGADCg_SDH2-1	C-term AD fusion for Y2H
At5g40650	SDH2-2	pDONR207	pENTR_SDH2-2	pK7FWG2	pKF_SDH2-2	C-term GFP fusion for AP-MS
At5g40650	SDH2-2	pDONR207	pENTR_SDH2-2	pDuExAc6	pDuExAc_SDH2-2	C-term N-Luc fusion for split-LUC

At5g40650	SDH2-2	pDONR207	pENTR_SDH2-2	pDuExDc6	pDuExDc_SDH2-2	C-term C-Luc fusion for split-LUC
At5g40650	SDH2-2	pDONR207	pENTR_SDH2-2	pGBKCg	pGBKCg_SDH2-2	C-term DBD fusion for Y2H
At5g40650	SDH2-2	pDONR207	pENTR_SDH2-2	pGADCg	pGADCg_SDH2-2	C-term AD fusion for Y2H
At5G65165	SDH2-3	pDONR207	pENTR_SDH2-3	pK7FWG2	pKF_SDH2-3	C-term GFP fusion for AP-MS
At5G65165	SDH2-3	pDONR207	pENTR_SDH2-3	pDuExAc6	pDuExAc_SDH2-3	C-term N-Luc fusion for split-LUC
At5G65165	SDH2-3	pDONR207	pENTR_SDH2-3	pDuExDc6	pDuExDc_SDH2-3	C-term C-Luc fusion for split-LUC
At5G65165	SDH2-3	pDONR207	pENTR_SDH2-3	pGBKCg	pGBKCg_SDH2-3	C-term DBD fusion for Y2H
At5G65165	SDH2-3	pDONR207	pENTR_SDH2-3	pGADCg	pGADCg_SDH2-3	C-term AD fusion for Y2H
At5g09600	SDH3-1	pDONR207	pENTR_SDH3-1	pK7FWG2	pKF_SDH3-1	C-term GFP fusion for AP-MS
At5g09600	SDH3-1	pDONR207	pENTR_SDH3-1	pDuExAc6	pDuExAc_SDH3-1	C-term N-Luc fusion for split-LUC
At5g09600	SDH3-1	pDONR207	pENTR_SDH3-1	pDuExDc6	pDuExDc_SDH3-1	C-term C-Luc fusion for split-LUC
At5g09600	SDH3-1	pDONR207	pENTR_SDH3-1	pGBKCg	pGBKCg_SDH3-1	C-term DBD fusion for Y2H
At5g09600	SDH3-1	pDONR207	pENTR_SDH3-1	pGADCg	pGADCg_SDH3-1	C-term AD fusion for Y2H
At2g46505	SDH4	pDONR207	pENTR_SDH4	pK7FWG2	pKF_SDH4	C-term GFP fusion for AP-MS
At2g46505	SDH4	pDONR207	pENTR_SDH4	pDuExAc6	pDuExAc_SDH4	C-term N-Luc fusion for split-LUC
At2g46505	SDH4	pDONR207	pENTR_SDH4	pDuExDc6	pDuExDc_SDH4	C-term C-Luc fusion for split-LUC
At2g46505	SDH4	pDONR207	pENTR_SDH4	pGBKCg	pGBKCg_SDH4	C-term DBD fusion for Y2H
At2g46505	SDH4	pDONR207	pENTR_SDH4	pGADCg	pGADCg_SDH4	C-term AD fusion for Y2H
At1g47420	SDH5	pDONR207	pENTR_SDH5	pK7FWG2	pKF_SDH5	C-term GFP fusion for AP-MS
At1g47420	SDH5	pDONR207	pENTR_SDH5	pDuExAc6	pDuExAc_SDH5	C-term N-Luc fusion for split-LUC
At1g47420	SDH5	pDONR207	pENTR_SDH5	pDuExDc6	pDuExDc_SDH5	C-term C-Luc fusion for split-LUC
At1g47420	SDH5	pDONR207	pENTR_SDH5	pGBKCg	pGBKCg_SDH5	C-term DBD fusion for Y2H
At1g47420	SDH5	pDONR207	pENTR_SDH5	pGADCg	pGADCg_SDH5	C-term AD fusion for Y2H
At1g08480	SDH6	pDONR207	pENTR_SDH6	pK7FWG2	pKF_SDH6	C-term GFP fusion for AP-MS
At1g08480	SDH6	pDONR207	pENTR_SDH6	pDuExAc6	pDuExAc_SDH6	C-term N-Luc fusion for split-LUC
At1g08480	SDH6	pDONR207	pENTR_SDH6	pDuExDc6	pDuExDc_SDH6	C-term C-Luc fusion for split-LUC
At1g08480	SDH6	pDONR207	pENTR_SDH6	pGBKCg	pGBKCg_SDH6	C-term DBD fusion for Y2H
At1g08480	SDH6	pDONR207	pENTR_SDH6	pGADCg	pGADCg_SDH6	C-term AD fusion for Y2H
At3g47833	SDH7a	pDONR207	pENTR_SDH7a	pK7FWG2	pKF_SDH7a	C-term GFP fusion for AP-MS
At3g47833	SDH7a	pDONR207	pENTR_SDH7a	pDuExAc6	pDuExAc_SDH7a	C-term N-Luc fusion for split-LUC
At3g47833	SDH7a	pDONR207	pENTR_SDH7a	pDuExDc6	pDuExDc_SDH7a	C-term C-Luc fusion for split-LUC
At3g47833	SDH7a	pDONR207	pENTR_SDH7a	pGBKCg	pGBKCg_SDH7a	C-term DBD fusion for Y2H
At3g47833	SDH7a	pDONR207	pENTR_SDH7a	pGADCg	pGADCg_SDH7a	C-term AD fusion for Y2H
At5g62575	SDH7b	pDONR207	pENTR_SDH7b	pK7FWG2	pKF_SDH7b	C-term GFP fusion for AP-MS
At5g62575	SDH7b	pDONR207	pENTR_SDH7b	pDuExAc6	pDuExAc_SDH7b	C-term N-Luc fusion for split-LUC
At5g62575	SDH7b	pDONR207	pENTR_SDH7b	pDuExDc6	pDuExDc_SDH7b	C-term C-Luc fusion for split-LUC
At5g62575	SDH7b	pDONR207	pENTR_SDH7b	pGBKCg	pGBKCg_SDH7b	C-term DBD fusion for Y2H
At5g62575	SDH7b	pDONR207	pENTR_SDH7b	pGADCg	pGADCg_SDH7b	C-term AD fusion for Y2H
At2g46390	SDH8	pDONR207	pENTR_SDH8	pK7FWG2	pKF_SDH8	C-term GFP fusion for AP-MS
At2g46390	SDH8	pDONR207	pENTR_SDH8	pDuExAc6	pDuExAc_SDH8	C-term N-Luc fusion for split-LUC
At2g46390	SDH8	pDONR207	pENTR_SDH8	pDuExDc6	pDuExDc_SDH8	C-term C-Luc fusion for split-LUC
At2g46390	SDH8	pDONR207	pENTR_SDH8	pGBKCg	pGBKCg_SDH8	C-term DBD fusion for Y2H
At2g46390	SDH8	pDONR207	pENTR_SDH8	pGADCg	pGADCg_SDH8	C-term AD fusion for Y2H
At2g47510	FUM1	pDONR207	pENTR_FUM1	pK7FWG2	pKF_FUM1	C-term GFP fusion for AP-MS
At2g47510	FUM1	pDONR207	pENTR_FUM1	pDuExAc6	pDuExAc_FUM1	C-term N-Luc fusion for split-LUC

At2g47510	FUM1	pDONR207	pENTR_FUM1	pDuExDc6	pDuExDc_FUM1	C-term C-Luc fusion for split-LUC
At2g47510	FUM1	pDONR207	pENTR_FUM1	pGBKCg	pGBKCg_FUM1	C-term DBD fusion for Y2H
At2g47510	FUM1	pDONR207	pENTR_FUM1	pGADCg	pGADCg_FUM1	C-term AD fusion for Y2H
At2g47510	FUM1	pDONR207	pENTR_FUM1	pDuScyCE	pDuScyCE_FUM1	C-term C-SCF fusion for BiFC
At1G53240	MDH1	pDONR207	pENTR_MDH1	pK7FWG2	pKF_MDH1	C-term GFP fusion for AP-MS
At1G53240	MDH1	pDONR207	pENTR_MDH1	pDuExAc6	pDuExAc_MDH1	C-term N-Luc fusion for split-LUC
At1G53240	MDH1	pDONR207	pENTR_MDH1	pDuExDc6	pDuExDc_MDH1	C-term C-Luc fusion for split-LUC
At1G53240	MDH1	pDONR207	pENTR_MDH1	pGBKCg	pGBKCg_MDH1	C-term DBD fusion for Y2H
At1G53240	MDH1	pDONR207	pENTR_MDH1	pGADCg	pGADCg_MDH1	C-term AD fusion for Y2H
At1G53240	MDH1	pDONR207	pENTR_MDH1	pDuVyNE	pDuVyNE_MDH1	C-term N-Venus fusion for BiFC
At3G15020	MDH2	pDONR207	pENTR_MDH2	pK7FWG2	pKF_MDH2	C-term GFP fusion for AP-MS
At3G15020	MDH2	pDONR207	pENTR_MDH2	pDuExAc6	pDuExAc_MDH2	C-term N-Luc fusion for split-LUC
At3G15020	MDH2	pDONR207	pENTR_MDH2	pDuExDc6	pDuExDc_MDH2	C-term C-Luc fusion for split-LUC
At3G15020	MDH2	pDONR207	pENTR_MDH2	pGBKCg	pGBKCg_MDH2	C-term DBD fusion for Y2H
At3G15020	MDH2	pDONR207	pENTR_MDH2	pGADCg	pGADCg_MDH2	C-term AD fusion for Y2H
At3G15020	MDH2	pDONR207	pENTR_MDH2	pDuVyNE	pDuVyNE_MDH2	C-term N-Venus fusion for BiFC
At2g13560	ME1	pDONR207	pENTR_ME1	pK7FWG2	pKF_ME1	C-term GFP fusion for AP-MS
At2g13560	ME1	pDONR207	pENTR_ME1	pDuExAc6	pDuExAc_ME1	C-term N-Luc fusion for split-LUC
At2g13560	ME1	pDONR207	pENTR_ME1	pDuExDc6	pDuExDc_ME1	C-term C-Luc fusion for split-LUC
At2g13560	ME1	pDONR207	pENTR_ME1	pGBKCg	pGBKCg_ME1	C-term DBD fusion for Y2H
At2g13560	ME1	pDONR207	pENTR_ME1	pGADCg	pGADCg_ME1	C-term AD fusion for Y2H
At2g13560	ME1	pDONR207	pENTR_ME1	pDuVyNE	pDuVyNE_ME1	C-term N-Venus fusion for BiFC
At4g00570	ME2	pDONR207	pENTR_ME2	pK7FWG2	pKF_ME2	C-term GFP fusion for AP-MS
At4g00570	ME2	pDONR207	pENTR_ME2	pDuExAc6	pDuExAc_ME2	C-term N-Luc fusion for split-LUC
At4g00570	ME2	pDONR207	pENTR_ME2	pDuExDc6	pDuExDc_ME2	C-term C-Luc fusion for split-LUC
At4g00570	ME2	pDONR207	pENTR_ME2	pGBKCg	pGBKCg_ME2	C-term DBD fusion for Y2H
At4g00570	ME2	pDONR207	pENTR_ME2	pGADCg	pGADCg_ME2	C-term AD fusion for Y2H
At4g00570	ME2	pDONR207	pENTR_ME2	pDuVyNE	pDuVyNE_ME2	C-term N-Venus fusion for BiFC

List of the expression plasmids constructed in this study. The full-length coding sequences of the listed genes (AGI code, without the stop codon) were cloned into pDONR207 Donor vector to generate Entry vectors (Entry vector). "Abbreviation" shows the name of enzyme proteins used in the text. Entry vectors were subjected to LR reactions with listed "Destination vector" to generate the "Expression vector" for the experiment mentioned on the "Purpose" column.

Supplementary Table 7: Ratio of isotopologues of product molecules during the isotope dilution experiments

						Ratio of isotopologues Time (min)											
Fig.	Substrate	Unlabelled Intermediate	Measured	Rep.	Isotopologue	0	20	40	60	70	80	85	90	95	100	105	110
4b	^{13}C-pyruvate	citrate	succinate	1	m+0		0.211	0.1034	0.0792	0.076	0.0721	0.0783	0.0723	0.0739	0.0709	0.0716	0.0754
					m+1		0.7203	0.8555	0.8839	0.8898	0.8919	0.8801	0.8918	0.8863	0.8951	0.8918	0.8859
					m+2		0.04	0.0322	0.0314	0.0308	0.031	0.0329	0.0315	0.0327	0.0314	0.0317	0.0329
					m+3		0.0105	0.0028	0.0016	0.0011	0.0017	0.0026	0.0013	0.0023	0.0003	0.0016	0.0016
					m+4		0.0182	0.0061	0.0039	0.0023	0.0033	0.0061	0.0031	0.0048	0.0023	0.0034	0.0043
4b	^{13}C-pyruvate	citrate	succinate	2	m+0		0.2829	0.1244	0.1126	0.1051	0.1156	0.1223	0.1221	0.1161	0.1118	0.1164	0.1085
					m+1		0.6777	0.8444	0.8522	0.8603	0.8472	0.8368	0.8408	0.846	0.852	0.8422	0.8569
					m+2		0.0271	0.0286	0.0312	0.0316	0.0324	0.0334	0.0324	0.0335	0.0328	0.0343	0.033
					m+3		0.0034	0.0004	0.0011	0.0008	0.0015	0.0026	0.0013	0.0013	0.0009	0.0025	0.0005
					m+4		0.0089	0.0022	0.003	0.0022	0.0033	0.0049	0.0035	0.0031	0.0024	0.0046	0.0012
4c	^{13}C-pyruvate	2-oxoglutarate	succinate	1	m+0*		0.134	0.1724	0.1063	0.0882		0.1147		0.1028	0.1353	0.1336	0.1287
					m+1		0.866	0.8276	0.8937	0.9118		0.8853		0.8972	0.8647	0.8664	0.8713
					m+2												
					m+3												
					m+4												
4c	^{13}C-pyruvate	2-oxoglutarate	succinate	2	m+0		0.2387	0.1357	0.1012	0.0934	0.0892	0.0999	0.1083	0.1118	0.1138	0.1135	0.1149
					m+1		0.6804	0.8127	0.8479	0.8589	0.8708	0.8626	0.8541	0.8467	0.8451	0.8505	0.8498
					m+2		0.0437	0.0348	0.0355	0.0349	0.0322	0.0309	0.0306	0.0327	0.0322	0.0308	0.0304
					m+3		0.0141	0.0057	0.0054	0.0043	0.0025	0.0021	0.0021	0.0027	0.0033	0.0018	0.0017
					m+4		0.0231	0.0111	0.0099	0.0085	0.0053	0.0045	0.0049	0.0061	0.0056	0.0034	0.0033
4e	^{13}C-glutamate	succinate	citrate	1	m+0		0.4284	0.1591		0.105	0.0807	0.0858	0.0956	0.0878	0.1383	0.1391	
					m+1		0.4925	0.775		0.8366	0.8692	0.8607	0.8568	0.8636	0.814	0.8152	
					m+2		0.0303	0.0355		0.0366	0.0374	0.0377	0.0362	0.0365	0.0351	0.0346	
					m+3		0.0162	0.0136		0.0108	0.0068	0.0074	0.0061	0.0064	0.0066	0.0059	
					m+4		0.0183	0.01		0.0068	0.0035	0.0051	0.0032	0.0035	0.0036	0.0031	
					m+5		0.0144	0.0069		0.0043	0.0024	0.0033	0.0021	0.0022	0.0024	0.002	
4e	^{13}C-glutamate	succinate	citrate	2	m+0		0.7226	0.1109	0.2493	0.0593	0.0501	0.0547	0.0562	0.0612	0.0853	0.0555	
					m+1		0.2409	0.7994	0.6944	0.888	0.8977	0.8938	0.8928	0.8856	0.8651	0.8957	
					m+2		0.0156	0.0418	0.033	0.0388	0.0396	0.0395	0.039	0.0389	0.04	0.0387	
					m+3		0.0051	0.0161	0.0079	0.0066	0.0065	0.0058	0.0059	0.0081	0.0059	0.0057	
					m+4		0.0097	0.0203	0.0097	0.0046	0.0037	0.0038	0.0037	0.0037	0.0022	0.0026	
					m+5		0.0061	0.0115	0.0058	0.0029	0.0024	0.0024	0.0024	0.0025	0.0015	0.0018	
4f	^{13}C-glutamate	fumarate	citrate	1	m+0		0.1607	0.3557	0.2288	0.0815	0.0577		0.0705	0.0677	0.0881	0.0515	0.0709
					m+1		0.7267	0.5891	0.7195	0.8567	0.8838		0.8704	0.8732	0.8579	0.8978	0.876
					m+2		0.0457	0.0306	0.0333	0.04	0.04		0.0409	0.0413	0.0405	0.0419	0.041
					m+3		0.0218	0.0096	0.0083	0.0098	0.0092		0.0086	0.009	0.0071	0.0052	0.0066
					m+4		0.0261	0.0085	0.0059	0.0071	0.0054		0.0058	0.0052	0.0037	0.002	0.003

					m+5		0.019	0.0065	0.0041	0.0049	0.0039		0.0038	0.0037	0.0027	0.0016	0.0025
4f	^{13}C-glutamate	fumarate	citrate	2	m+0	0.7134	0.2993	0.0667	0.0972	0.0783	0.062	0.0627	0.0576	0.0542	0.0546	0.054	0.0754
					m+1	0.0981	0.6146	0.8792	0.851	0.8724	0.8883	0.8887	0.8945	0.8976	0.8981	0.8992	0.8666
					m+2	0.0498	0.0365	0.039	0.0374	0.0378	0.0387	0.0382	0.0384	0.0388	0.0387	0.0396	0.0406
					m+3	0.0326	0.014	0.007	0.0065	0.0052	0.0053	0.0056	0.0052	0.0051	0.0048	0.0046	0.0076
					m+4	0.0622	0.0203	0.0047	0.0045	0.0036	0.0033	0.0028	0.0023	0.0025	0.0023	0.0015	0.0056
					m+5	0.0441	0.0154	0.0033	0.0034	0.0026	0.0023	0.0019	0.0019	0.0017	0.0016	0.0011	0.0042
4g	^{13}C-glutamate	malate	citrate	1	m+0					0.0663	0.0504	0.0534	0.0534	0.0581		0.0546	0.0564
					m+1					0.8416	0.8859	0.8865	0.8865	0.8852		0.8881	0.889
					m+2					0.0454	0.0422	0.0411	0.0411	0.0403		0.0419	0.0418
					m+3					0.016	0.0095	0.0083	0.0083	0.0074		0.0073	0.0062
					m+4					0.0178	0.0069	0.0059	0.0059	0.0053		0.0045	0.0036
					m+5					0.0129	0.0052	0.0047	0.0047	0.0037		0.0037	0.003
4g	^{13}C-glutamate	malate	citrate	2	m+0					0.0605	0.0543	0.0543	0.0602	0.0639	0.0761	0.0689	0.075
					m+1					0.8825	0.8907	0.8897	0.8869	0.8853	0.8724	0.8834	0.8725
					m+2					0.0402	0.04	0.0391	0.0384	0.0386	0.0377	0.0383	0.0387
					m+3					0.0068	0.0074	0.0081	0.0072	0.0067	0.0073	0.0053	0.0077
					m+4					0.0059	0.0048	0.0055	0.0045	0.0028	0.004	0.0022	0.0039
					m+5					0.004	0.0028	0.0033	0.0029	0.0027	0.0024	0.0018	0.0022

The fragments of succinate and citrate used for evaluation of ^{13}C enrichment contain 4 and 5 carbons, respectively. The ratio of individual isotopologues at each time points in duplicated experiments is shown.

*, The abundance of M+0 isotopologue is calculated from that of M+1 with an assumption of absence of M+2, M+3 and M+4 due to the lack of data other than the abundance of M+1.
